# Investigations of metastable Ca_2_IrO_4_ epitaxial thin-films: systematic comparison with Sr_2_IrO_4_ and Ba_2_IrO_4_

**DOI:** 10.1038/srep25967

**Published:** 2016-05-19

**Authors:** M. Souri, J. H. Gruenewald, J. Terzic, J. W. Brill, G. Cao, S. S. A. Seo

**Affiliations:** 1 Department of Physics and Astronomy, University of Kentucky, Lexington, KY 40506, USA

## Abstract

We have synthesized thermodynamically metastable Ca_2_IrO_4_ thin-films on YAlO_3_ (110) substrates by pulsed laser deposition. The epitaxial Ca_2_IrO_4_ thin-films are of K_2_NiF_4_-type tetragonal structure. Transport and optical spectroscopy measurements indicate that the electronic structure of the Ca_2_IrO_4_ thin-films is similar to that of *J*_eff_ = 1/2 spin-orbit-coupled Mott insulator Sr_2_IrO_4_ and Ba_2_IrO_4_, with the exception of an increased gap energy. The gap increase is to be expected in Ca_2_IrO_4_ due to its increased octahedral rotation and tilting, which results in enhanced electron-correlation, *U*/*W*. Our results suggest that the epitaxial stabilization growth of metastable-phase thin-films can be used effectively for investigating layered iridates and various complex-oxide systems.

The spin-orbit assisted Mott state discovered in layered iridates, e.g. Sr_2_IrO_4_, provides a new platform to realize unconventional properties of condensed matter due to the unique coexistence of strong spin-orbit coupling and electron-correlation^1^. Recent studies have revealed the possibilities of novel electronic and magnetic phases in iridates such as Weyl semimetals^2^,^3^, and a potential high-*T*_c_ superconducting state with *d*-wave gap^4^,^5^,^6^,^7^. However, the fundamental electronic structure of the layered iridate is still under debate; namely, the insulating gap may open due to antiferromagnetic ordering, i.e. Slater scheme^8^,^9^, rather than electron-correlation, i.e. Mott picture. Moreover, it is a formidable task to unveil the physics of layered iridates since only Sr_2_IrO_4_ and Ba_2_IrO_4_ (refs 10, 11, 12, 13) phases are available for experimental characterizations to date.

In this article, we report the systematic changes of the structural, transport, and optical properties of layered iridates by investigating meta-stable Ca_2_IrO_4_ epitaxial thin-films. Since the Ruddlesden-Popper (*R*-*P*) phase of Ca_2_IrO_4_ is not thermodynamically stable, its bulk crystals do not exist in nature. However, we have successfully synthesized the *R*-*P* phase Ca_2_IrO_4_ thin-films (Fig. 1(b)) from a polycrystalline hexagonal (*P62m*) Ca_2_IrO_4_ bulk crystal (Fig. 1(a)) using an epitaxial stabilization technique^14^. The smaller ionic size of Ca^2+^ compared to Sr^2+^ causes increased IrO_6_ octahedral rotation and/or tilting, hence a reduced electronic band-width (*W*). Thus, investigating Ca_2_IrO_4_ in a comparative study with Sr_2_IrO_4_ and Ba_2_IrO_4_ provides a unique opportunity to explore the layered iridate system, as it allows for the enhancement of the electronic correlation effect (*U*/*W*).

## Methods

**Figure 1:**
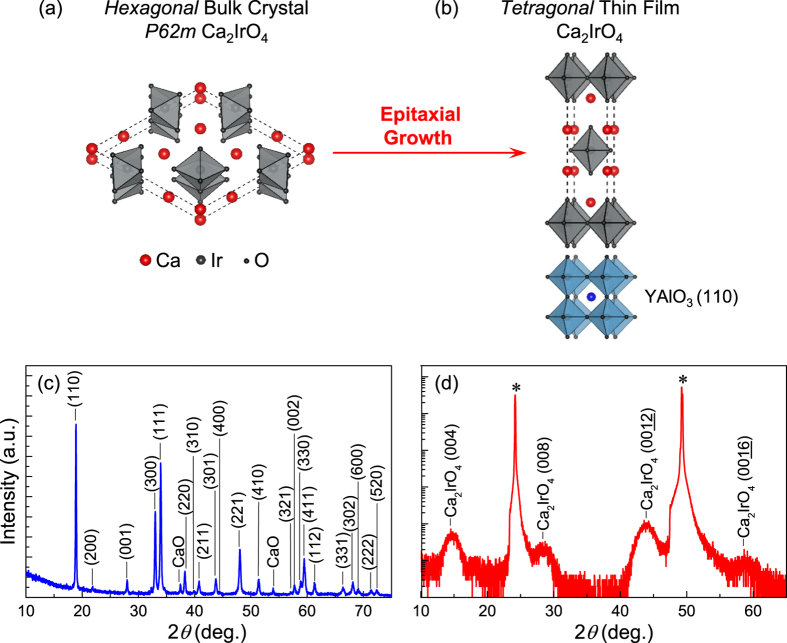
Schematic diagram of epitaxial stabilization of tetragonal Ca_2_IrO_4_ epitaxial thin-film from (**a**) the bulk hexagonal phase of Ca_2_IrO_4_, i.e. a target used in the pulsed laser deposition, to (**b**) metastable *R-P* phase of Ca_2_IrO_4_ thin-film grown on a single crystal YAlO_3_ (110) substrate. (**c**) Powder x-ray diffraction of our target material, which shows x-ray diffraction peaks from the hexagonal bulk phase of *P62m* and a couple of small CaO peaks. (d) X-ray 2*θ-ω* scan of an epitaxial Ca_2_IrO_4_ thin-film, where only the (00*l*)-diffraction peaks of Ca_2_IrO_4_ are visible. YAlO_3_ (110) and (220) peaks are labeled with asterisk (*) symbols.

We have grown metastable Ca_2_IrO_4_ epitaxial thin-films with the K_2_NiF_4_–type crystal structure on YAlO_3_ (110) substrates by using a custom-built pulsed laser deposition (PLD) system with *in-situ* spectroscopic monitoring techniques^15^. The laser ablation is performed on a polycrystalline hexagonal (*P62m*) Ca_2_IrO_4_ target. The powder x-ray diffraction of the target is presented in Fig. 1(c). The samples are grown under the growth conditions of 1.2 J/cm^2^ laser fluence (KrF excimer, *λ* = 248 nm), and 700 °C substrate temperature. In order to avoid defects such as oxygen vacancies during the growth, we have used a laser beam imaging technique with reduced laser beam size in PLD to minimize the kinetic energy of the plume^16^. This technique is essential for the successful growth of Ca_2_IrO_4_ thin-films. A relative high oxygen partial pressure of 10 mTorr is also used to minimize oxygen vacancies. The structural properties of the epitaxial Ca_2_IrO_4_ thin-films are measured using x-ray diffractometry (Bruker D8 Advance system with Cu-Ka radiation). Transport properties are measured using a Physical Property Measurement System (Quantum Design) with conventional four-probe and Hall geometries. Optical transmission spectra (*T*(*ω*)) are taken at normal incidence using a Fourier-transform infrared spectrometer in the photon energy region of 0.2–0.6 eV and a grating-type spectrophotometer in the range of 0.5–7 eV, where the substrates are transparent. The absorption spectra are calculated using 
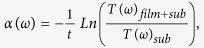
 where *t* is the thin film thickness.

## Results and Discussion

**Figure 2:**
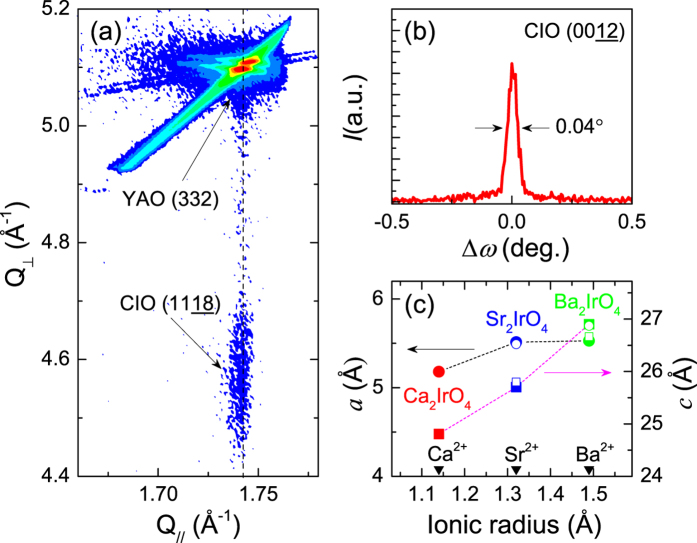
(**a**) Reciprocal space map near the YAlO_3_ (332)-reflection, which shows the Ca_2_IrO_4_ (1118)-reflection. The vertical dashed line indicates that the Ca_2_IrO_4_ thin-film is coherently strained to the substrate. (**b**) The rocking curve scan of Ca_2_IrO_4_ (0012)-reflection has a full-width half-maximum of 0.04°. (**c**) The in-plane (left axis) and out of plane (right axis) lattice parameters of Ca_2_IrO_4_, Sr_2_IrO_4_ (ref. 17) and Ba_2_IrO_4_ (ref. 10) thin films obtained from x-ray diffraction scans, as a function of A-site cation ionic radius. The solid circles and squares present the in-plane and out of plane lattice parameters, respectively. The open symbols indicate the in-plane and out of plane lattice parameters of Sr_2_IrO_4_ and Ba_2_IrO_4_ single crystals^12^,^23^,^24^.

The metastable *R-P* phase of the Ca_2_IrO_4_ thin films is verified by x-ray diffraction and reciprocal space mapping scans, which indicate that the films are stabilized by the epitaxial strain of the substrates and are of high crystalline quality. Figure 1(d) shows the *θ*-2*θ* x-ray diffraction scan with the (00l) peaks of a Ca_2_IrO_4_ thin film. The full width at half maximum of the (0012)-reflection rocking curve scan is 0.04°, which clearly shows good crystallinity of the film (Fig. 2(b)). The thickness of the Ca_2_IrO_4_ thin films is ca. 6 nm. The crystal quality deteriorates considerably as we increase the thickness further, presumably due to its thermodynamically metastable nature. In x-ray reciprocal space mapping (Fig. 2(a)), the (1118) peak of the film is vertically aligned with the YAlO_3_ substrate (332)-reflection, indicating Ca_2_IrO_4_ films are coherently strained to the substrates, i.e. [110]_film_//[001]_substrate_ and [001]_film_//[110]_substrate_. The lattice parameters obtained from the x-ray diffraction scans show that both in-plane (*a*) and out-of-plane (*c*) lattice parameters of Ca_2_IrO_4_ films are smaller than those of Sr_2_IrO_4_ (ref. 17) and Ba_2_IrO_4_ (ref. 10) (Fig. 2(c)). At this moment, the local structural information of Ca_2_IrO_4_ films, such as octahedral rotation and tilting, is unknown and requires substantial microscopic characterizations that we plan to perform as a future study. However, by assuming the rigid Ir-O bond-length to be constant, which is a reasonable assumption, we conjecture the reduced lattice constants (from x-ray diffraction) imply that the Ir-O-Ir bond angle is reduced from 158° (Sr_2_IrO_4_) to ca. 140° (Ca_2_IrO_4_). The reduced bond angle implies a corresponding reduction in bandwidth (*W*), according to the relation between bandwidth (*W*) and the Ir-O-Ir bond angle (*θ*) described by^18^:


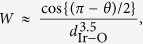


where *d*_Ir-O_ is the Ir-O bond length. This will result in an enhanced electron-correlation (*U*/*W*) for the Ca_2_IrO_4_ compound as compared to that of the Sr_2_IrO_4_ and Ba_2_IrO_4_ thin films.

**Figure 3:**
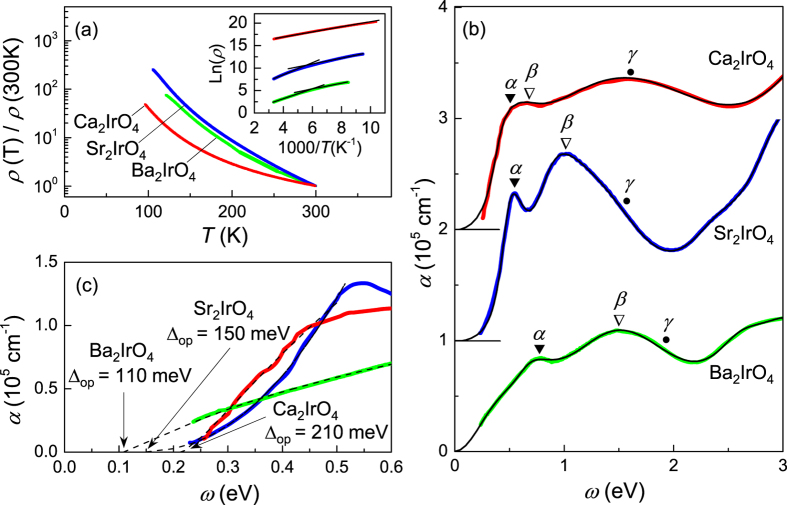
(**a**) Normalized resistivity (*ρ*) versus temperature data of Ca_2_IrO_4_ (red), Sr_2_IrO_4_ (blue) and Ba_2_IrO_4_ (green) thin-films. The data of Sr_2_IrO_4_ is from ref. 10; The inset shows the Arrhenius plot of Ca_2_IrO_4_, Sr_2_IrO_4_ and Ba_2_IrO_4_. Solid black lines present the linear fits at room temperature and low temperature. The estimated gap energies at room temperature are Δ_CIO_ = 120 meV, Δ_SIO_ = 250 meV, and Δ_BIO_ = 190 meV. The Arrhenius plots are shifted vertically for clarity. (**b**) Optical absorption spectra (*α* (ω)) of Ca_2_IrO_4_, Sr_2_IrO_4_ and Ba_2_IrO_4_ thin-films at room temperature. The plots are shifted vertically by 10^5^ cm^−1^ for clarity. The *α, β* and *γ* represent the optical transition peak energies obtained from the fit with the minimal set of the Lorentz oscillators. The solid black curves are the fit curves using Lorentz oscillators, which match well with the experimental spectra. (**c**) Fitted absorption spectra of Ca_2_IrO_4_, Sr_2_IrO_4_ and Ba_2_IrO_4_ at low energy using Wood-Tauc’s method^21^ which clearly confirm the increased energy gap from Ba_2_IrO_4_ to Ca_2_IrO_4_. The estimated optical gap energies using this method are Δ_CIO_ = 210 meV, Δ_SIO_ = 150 meV, and Δ_BIO_ = 110 meV.

Figure 3(a) shows the temperature-dependent resistivity *ρ*(T) of a Ca_2_IrO_4_ thin film, which has an insulating behavior. The room-temperature resistivity of Ca_2_IrO_4_ (ca. 170 mΩcm) is about the same as the room temperature resistivity of Sr_2_IrO_4_ (ca. 140 mΩcm) and Ba_2_IrO_4_ (ca. 130 mΩcm) deposited on SrTiO_3_ substrates. The energy gap (Δ = 2*E*_a_) of Ca_2_IrO_4_ is calculated using an Arrhenius plot (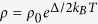, where *k*_*B*_ is the Boltzmann constant) and compared to Sr_2_IrO_4_ (ref. 10) and Ba_2_IrO_4_ thin films. While the Arrhenius plots of Sr_2_IrO_4_ (ref. 10) and Ba_2_IrO_4_ show non-linear behaviors, the transport of Ca_2_IrO_4_ is quite linear over the entire measured temperature range (300 K to 90 K). An energy gap of 120 meV is extracted from its Arrhenius plot. Due to the increased *U*/*W* in Ca_2_IrO_4_, we expect its gap energy to be larger than that of Ba_2_IrO_4_ and Sr_2_IrO_4_. However, the energy gap of Ca_2_IrO_4_ obtained from the room temperature transport is smaller than that of Sr_2_IrO_4_ and Ba_2_IrO_4_. This puzzling observation implies that the transport properties of layered iridates are mostly dominated by impurities or defects, and intrinsic bandgap energies should be measured using a spectroscopic technique.

Figure 3(b) presents the optical absorption spectra (*α*(*ω*)) of Ca_2_IrO_4_ compared with Sr_2_IrO_4_ (ref. 17) and Ba_2_IrO_4_ (ref. 10) thin films. The absorption spectra are fit using a minimal set of Lorentz oscillators. The common features of strong absorption tails due to the charge-transfer transitions from O 2*p* to Ir 5*d* bands are above ca. 2–3 eV. The black solid lines in Fig. 3(b) are the resultant fit curves using Lorentz oscillators, which match well with the experimental spectra. The three absorption peaks indicated by *α, β*, and *γ* are labeled consistently with previous literature^19^,^20^. The *α, β*, and *γ* absorption bands have been interpreted as the associated Ir 5*d* transitions, such as Ir-Ir intersite optical transitions^1^,^19^,^20^. Note that as the cation size — and consequently the Ir 5*d* bandwidth — increases from Ca_2_IrO_4_ to Ba_2_IrO_4_, the *α, β,* and *γ* peak-positions are shifted to *higher* energy. This seemingly counterintuitive peak shift has also been observed in the optical absorption spectra of strain-dependent Sr_2_IrO_4_ thin-films^17^, as the lattice strain changes from compressive to tensile directions. This observation of the peak-energy shift can provide a key to understanding the electronic structures of iridates since the spectral shape is thought to be strong experimental evidence supporting the Mott picture of this system^1^,^19^,^20^. However, we will leave it as a future study since detailed analysis requires theoretical modeling and calculations, which is beyond the scope of this article.

We note the increased optical gap energy of Ca_2_IrO_4_ thin-films as compared to that of Sr_2_IrO_4_ and Ba_2_IrO_4_. To calculate the optical energy gap, each absorption spectrum is fit using the Wood-Tauc’s method^21^ (Fig. 3(c)). In this method, the strong region of the absorption edge (α > 10^4^ cm^−1^) can be described by:


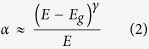


where *E*_*g*_ (*E*) is the optical band gap (incident photon) energy. The estimated optical gap energies using this method are Δ_CIO_ = 210 meV, Δ_SIO_ = 150 meV, and Δ_BIO_ = 110 meV. For the exponent *γ*, we have obtained *γ* = 1.5 (Ca_2_IrO_4_), *γ* = 3.0 (Sr_2_IrO_4_), and *γ* = 1.5 (Ba_2_IrO_4_). While *γ* = 3 is consistent with the indirect bandgap of Sr_2_IrO_4_, *γ* = 1.5 values in Ca_2_IrO_4_ and Ba_2_IrO_4_ suggests direct bandgap, of which physical understanding will require further theoretical studies. Nevertheless, as shown in Fig. 3(c), the optical gap energy has clearly increased for Ca_2_IrO_4_ compared to that of Sr_2_IrO_4_ and Ba_2_IrO_4_. Hence, as we decrease the ionic sizes of A-site cations in layered iridates, the Ir-O-Ir bond angle is reduced, which, in turn, increases *U/W* and manifests itself as the observed increase in the optical bandgap energy.

Our approach of synthesizing meta-stable phase thin-films of strongly correlated systems offers a new route to understanding the physics of complex oxides. For example, the stabilization of metastable phases can provide compounds with larger effective electronic correlations than presently available by producing increased distortion and tilting in lattice. While simple octahedral distortions usually preserve inversion symmetry in the K_2_NiF_4_–type structure, the *R*-*P* structure of Ca_2_IrO_4_ has been proposed as a candidate material featuring a non-centrosymmetric structure due to its low symmetry^22^. This unique structure, achieved by breaking the inversion symmetry in this system, is expected to induce many interesting phase transitions such as ferroelectricity and multiferroicity. Hence, experimental studies of meta-stable phases allow us to tackle a number of intriguing problems of exotic ground states with novel properties that are theoretically suggested.

## Conclusion

We have successfully stabilized Ca_2_IrO_4_ thin-films with the K_2_NiF_4_–type crystal structure and determined its higher optical gap energy to originate from its enhanced electron-correlation, *U*/*W*, with respect to its larger *A*-site cation isosymmetric compounds. The structural study confirms the good crystallinity and coherent strain state of the epitaxial Ca_2_IrO_4_ thin-films on YAlO_3_ (110) substrates. The transport and optical spectroscopic experiments show that Ca_2_IrO_4_ thin-films have an insulating ground state similar to Sr_2_IrO_4_ and Ba_2_IrO_4_. However, the increased IrO_6_ octahedral rotation, tilting, or distortion in Ca_2_IrO_4_ increases *U*/*W*, and thus its optical gap energy is larger than the gap energies of Sr_2_IrO_4_ and Ba_2_IrO_4_. This approach of metastable thin-film phases can greatly expand the number of available materials and can help to unveil the physics of strongly correlated systems.

## Additional Information

**How to cite this article**: Souri, M. *et al*. Investigations of metastable Ca_2_IrO_4_ epitaxial thin-films: systematic comparison with Sr_2_IrO_4_ and Ba_2_IrO_4_. *Sci. Rep.* **6**, 25967; doi: 10.1038/srep25967 (2016).
